# DDoS attack detection method based on improved convolutional long short-term memory and three-way decision in SDN

**DOI:** 10.1371/journal.pone.0322839

**Published:** 2025-05-14

**Authors:** Haizhen Wang, Xiaojing Yang, Na Jia

**Affiliations:** 1 College of Computer and Control Engineering, Qiqihar University, Qiqihar, China; 2 Heilongjiang Key Laboratory of Big Data Network Security Detection and Analysis, Qiqihar University, Qiqihar, China; University of Calabar, NIGERIA

## Abstract

Software Defined Networking (SDN) is an emerging network architecture and management method, whose core idea is to separate the network control plane from the data transmission plane. It is precisely because of this characteristic that SDN controllers are susceptible to external malicious attacks, the most common of which are Distributed Denial of Service (DDoS) attacks. This paper suggests a way to find DDoS attacks called ConvLTSM-MHA-TWD. It is based on the Convolutional Long Short-Term Memory Network (ConvLSTM) and three-way decision (TWD). It solves the problem of insufficient feature extraction in SDN environment and improves classification accuracy. This method uses ConvLSTM to extract data features, and uses multi-head attention (MHA) mechanism to learn the long-distance dependence relationship in the input data, and then constructs multi-granularity feature space. ConvLSTM and MHA outputs are added to form a residual connection to further enhance feature extraction and timing modeling capabilities and solve the problem of gradient disappearance during model training. Then the three-way decision theory is used to make decisions on network behaviors immediately. For the network behaviors that cannot be made immediately, the delayed decision is made, and the feature extraction and decision are made on this part of the network behaviors again. Finally, the classification results are output. This paper conducted experiments on data sets CICIDS2017 and DDoS SDN, with accuracy rates of 0.994 and 0.977, respectively, which has better overall performance, and is suitable for training large amounts of data.

## 1 Introduction

In today’s digital age, the Internet has become an integral part of our lives and business activities. Software-Defined Networking (SDN) [1–4] as a revolutionary network architecture, technology has changed the way traditional networks operate. SDN separates the network control plane from the data plane, allowing network administrators to manage and configure network traffic through a centralized controller. This architecture makes the network more flexible and programmable. However, this centralized management model makes SDN controllers the best targets for malicious attacks by intruders. Denial of service (DoS) attacks and distributed denial of service (DDoS) attacks [5] exhaust SDN controller computing resources, occupy network bandwidth and the upper limit of TCP connections, and slow down controller response until it finally fails to provide normal services. The security issues of SDN have also become more serious and complex, especially distributed denial of service (DDoS) attacks, which have become one of the major challenges in network threats, bringing great risks to network availability and data integrity. Traditional DDoS defense methods, such as hardware-based firewalls [6–7] and intrusion detection systems (IDS) [8], have been able to mitigate DDoS attacks to some extent, but they are clearly inadequate in the face of evolving attack techniques and attack scale. Therefore, there is an urgent need for smarter and more efficient solutions to deal with the complexity of DDoS attacks.

ConvLSTM [9] can capture spatiotemporal features, and TWD theory [10] improves classification accuracy by delaying decision-making. We propose ConvLSTM-MHA-TWD to detect DDoS attacks for SDN using improved ConvLSTM and TWD theory, The main contributions of this paper are as follows:

■ ConvLSTM is improved by using batch normalization to process convolutional output and using the ReLU activation function to nonlinearly transform the output result. The multi-head attention mechanism was added to help the model better capture the dependencies between temporal features.
■ The outputs of improved ConvLSTM and multi-head attention mechanisms are added together to form residual connections, blending local and global features and mitigating gradient disappearance. To avoid the risk of data misclassification, this paper introduces the three-way decision theory for classification.
■ We conducted experiments on the CIC-IDS2017 and DDoS SDN Data sets. The experimental results show that the proposed method has higher accuracy and F1 values than the comparison method.

## 2 Related works

The detection of DDoS attacks in traditional networks includes the use of classical machine learning methods [11]. However, the traditional machine learning algorithm is difficult to extract the features of the intrusion data and highly relies on feature selection. During model training, it is necessary to artificially design features to improve the model’s performance. The deep learning model [12] can automatically learn multi-level, high-level, and nonlinear feature representations from raw data without manually designing feature extractors. This eliminates the need for manual design feature engineering, is able to adapt to a variety of data types and domains, and has excellent generalization and adaptability. Because the current attack data has many characteristics, large data dimensions, and data redundancy, it is necessary to conduct dimensionality reduction processing during data preprocessing for better training. Ibor, A.E. et al. [13] divided attack data learning into two stages. In the first stage, feature engineering was carried out during unsupervised learning, and Principal Component Analysis(PCA) was used for feature screening to remove redundant information in the data and linear correlation between features. Abdulhammed, R et al. [14] used AE and PCA to reduce the dimension of the data when processing the data and then constructed various classifiers to detect the classification, and the detection result reached 99%. Salo, F et al. [15] proposed a new hybrid dimension reduction technique, combining information gain (IG) and PCA techniques to reduce data dimensions, and then using a set classifier based on SVM, IBK, and MLP algorithms for classification, achieving the best performance in terms of classification accuracy.

When classifying network data, the support vector machine method [16], Bayesian method [17], neural network method [18], cluster analysis method [19], artificial neural network method [20] and KNN method [21] are mostly used as traffic classifiers in an SDN environment. These methods use the traditional binary classification method when detecting network traffic. When faced with network behavior, the classification method can only take two possibilities, and there will be a lot of wrong classifications of data. Due to the influence of machine learning research and the development of deep learning, deep learning technology has been favored by many scholars in intrusion detection in recent years. Ge Jike et al. [22] proposed a network intrusion detection method that combines improved convolutional neural networks with long short-term memory networks (GCNN-LSTM). The model uses a global pooling layer instead of a full connection layer to improve CNN. Combined with the powerful time series learning ability of the LSTM algorithm, the data after feature selection is trained for classification and prediction. Bai Jianjing et al. [23] proposed a scheme to detect DDoS attacks in an SDN environment by using an LSTM network, a long short-term memory network. A lightweight neural network based on Bi-LSTM is deployed in the idle edge nodes of the Internet of Things to complete the detection task, which increases the flexibility of detection while ensuring accuracy. Al Razib et al. [24] proposed that DL model-driven SDN can significantly reduce the attacks faced by IDS in SDN. Zainudin et al. [25] proposed a low-cost DDoS attack classification method. The study combines CNN and LSTM to design extreme gradient enhancement. Alghazzawi et al. [26] proposed an effective hybrid DL model (CNN+BILSTM). The method establishes an X2 test for feature selection and then classifies DDoS attacks using a mixed CNN+BILSTM model. Javeed [27] developed an SDN-enabled DL driver solution using a hybrid technology, including CUDA-Deep Neural Network Short Term Memory (CUDNNLSTM) and CUDA-Deep Neural Network Gated Recursive Unit (CUDNNGRU) algorithms for effective threat detection.

The above studies all adopt the traditional binary classification and there is a problem of insufficient feature extraction, which leads to a high number of errors in classification, and thus affects the accuracy of detection. To address these problem, this paper presents a new DDoS attack method integrating ConLSTM and TWD.

## 3 The proposed method

### 3.1 Overall framework design

**Fig 1:**
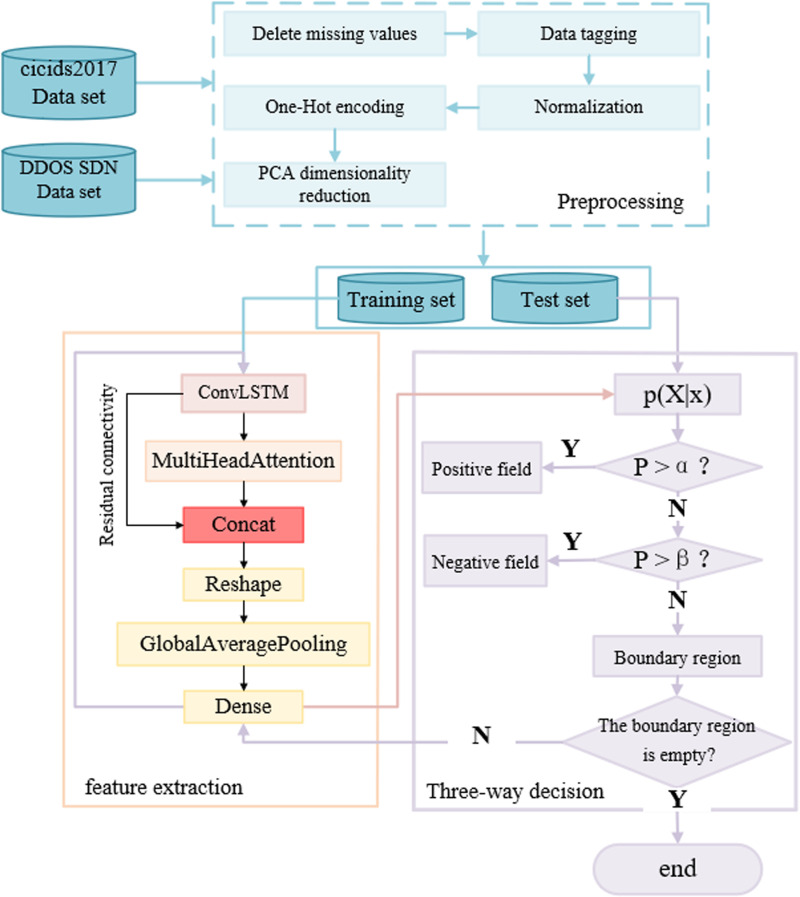
Flowchart of ConvLSTM-MHA-TWD.

The attack detection process of ConvLSTM-MHA-TWD is shown in Fig 1, which mainly includes three modules: preprocessing, feature extraction, and three-way decision through the improved ConvLSTM and TWD. The preprocessing data is first extracted by the improved ConvLSTM and then judged by three-way decisions. The probability of the data in the three-way decision modules belonging to the positive domain is first judged. If the condition is greater than α or less than β, the positive and negative domain are directly divided; if not, the positive and negative domain are divided into the boundary domain, and the data in the boundary domain is extracted again by the feature extraction module, and the decision is remade. When the data in the boundary domain re-enter the convolutional short-duration memory network for feature extraction, different features will be extracted based on the previous feature extraction, thus providing more data information for the classifier to re-classify, thus supporting the classifier to make decisions on samples in the boundary domain. The whole process will continue until there are no more samples in the boundary domain.

Below is a detailed introduction to the design of the three modules.

### 3.2 Preprocessing

Firstly, deleting the missing values and outliers in the original data set, then the normal data and attack data 0, 1 label, next, the features are normalized and encoded by unique heat. Finally, PCA is used for dimensionality reduction.

Due to PCA has high computational efficiency and strong interoperability, and the large amount of data and high data dimension in our work, PCA was selected for dimensionality reduction to preserve the global structure of the data, and then dimensionality reconstruction of the features after dimensionality reduction was carried out. Finally 30 dimensionality feature were transferred to the convolutional long-term memory network for feature extraction.

### 3.3 Feature extraction

**Fig 2:**
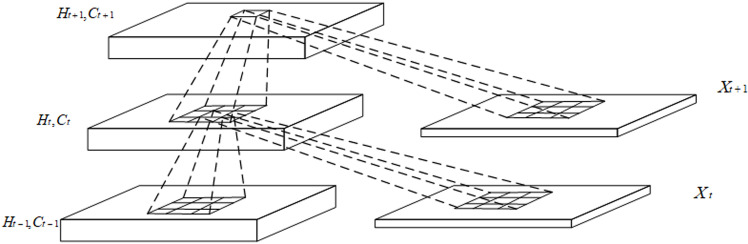
Internal structure of ConvLSTM.

When the convolutional long-duration memory network (the internal structure of ConvLSTM is shown in Fig 2) is used for feature extraction, the network is supervisedly trained. The convolutional layer is mainly used to capture the spatial features in the input sequence, the pooling layer is used to reduce the complexity of the model, and the long-duration memory layer is used to capture the time dependence in the sequence. In order to minimize the error between the model prediction and the actual label, the backpropagation algorithm is used to update the parameters iteratively during the training of the neural network.

**Fig 3:**
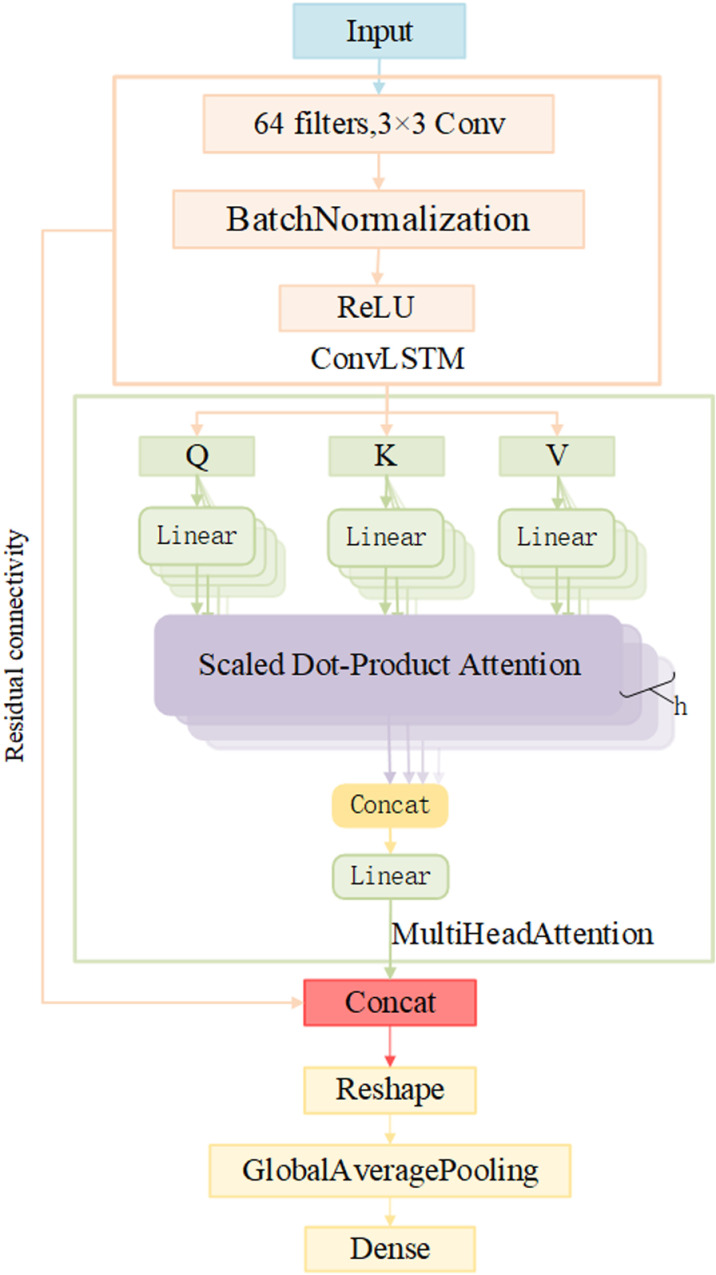
ConvLSTM structure based on residual connection.

In this paper, the improved ConvLSTM network (as shown in Fig 3) is used for feature extraction of input data.

The network structure uses the convolution operation in ConvLSTM and the time dependence of LSTM to capture the spatial and time series of data. In order to speed up the training speed and improve the training stability of the network, the convolutional output is processed by batch normalization. The ReLU activation function is used to transform the output nonlinearly. The output of the nonlinear transformation is used as the query, key, and value of the MHA mechanism, and the long-distance dependencies of the input data are learned through multiple attention heads, and the input is weighted from multiple perspectives, thus obtaining a richer and more comprehensive feature representation and further enhancing the ability of the model to capture complex dependencies in the data. The output of ConvLSTM and MHA is then combined to form a residual connection that can effectively fuse local and global features, capture multiple layers of information from the data to achieve deeper feature learning, and retain the original feature information to alleviate the gradient disappearance problem. Finally, the global average pooling layer is used to reduce the dimension of the output, which reduces the complexity of the model while preserving the important global information of the input data.

### 3.4 Three-way decision

#### 3.4.1 Algorithm description.

Suppose the sample set is $\text{x}=\{{\text{x}}_{1},{\text{x}}_{2},\ldots ,{\text{x}}_{\text{n}}\;\;\;\}\;\;\;$, POS is a positive field, NEG is a negative field and BND is the boundary field, the probability $\text{p}(\text{POS}|{\text{x}}_{\text{i}})$ that the sample $x_{i}$ belongs to the positive field needs to be solved, where i= 1,2,... , n. Compare the probability value p with the threshold values α, β: if p>α, it is divided into a positive field, if p<β, it is divided into a negative field, otherwise it is divided into a boundary field. Suppose that the original training set is $T_{r}$, the test set is $T_{e}$, and the classifier is f. The purpose of f is to make decisions on every sample of data in $T_{e}$ as accurately as possible. Suppose that the final decision set made by f on $T_{e}$ is Y, then Y = POS∪NEG. If Y is not the final decision set, then Y =POS∪NEG∪BND, that is, the set of sample data for delayed decision-taking. Before the final decision, BND =POS∪NEG∪BND, BND  is the new boundary field obtained on the basis of BND. The specific algorithm steps are as follows:

1: Three-way decision algorithms

2:   Input: training set $T_{r}$, test set $T_{e}$

3:   Output: positive field *POS*, negative field *NEG*

4:   1.Initialization parameters: ConvLSTM feature extraction mode *G*；Threshold

5:α,β；Initial classifier f;

6:   Positive domain（*POS*）= negative field（*NEG*）= Boundary field

7:（*BND*）=∅

8:   2. Do

9:   2.1. $T_{r}=G\left(T_{r}\right)$；$T_{e}=G\left(T_{e}\right)$;

10:  2.2. Train classifier f  according to $T_{r}$;

11:   2.3. The probability *P=*$f\left(T_{e}\right)$ that each data sample in $T_{r}$ belongs to a positive 12: class is obtained from model f

13:  2.4. For each p∈P，$t_{e}\in T_{e}$:

14:           If p>α:

15:              $POS=POS\cup T_{e}$;

16:           Else if p<β:

17:              $NEG=NEG\cup T_{e}$;

18:           Else:

19:              $BND=BND\cup T_{e}$;

20:           End

21:     End

22:      *G*(*BND*) → *T_e_*

23:  Until the test set $T_{e}$ is empty

24: 3. Output: POS∪NEG.

#### 3.4.2 Threshold value setting.

When the three-way decision theory is used to classify the data, the value of threshold pairs α and β is obtained from the loss function, and the positive domain, negative domain, and boundary domain are divided accordingly. Different loss functions correspond to different threshold pairs, and different threshold settings lead to different partition results. In this paper, the three-way decision theory is applied to the research of DDoS intrusion detection methods, which is mainly used to determine whether the network behavior belongs to normal behavior or attack behavior, and the choice of loss function is closely related to intrusion detection. According to expert experience, the cost of mistaking a normal network behavior for abnormal behavior is much lower than the cost of mistaking an abnormal network behavior for normal behavior. Therefore, various loss functions can be set up as shown in Table 1.

**Table 1:** Loss function values of the three-way decisions.

Decision	Accept	Refuse
***POS***	0.0	0.8
***NEG***	0.2	0.2
***BND***	1.0	0.0

Through the set experience value, the corresponding threshold can be calculated according to formulas (1) and (2).


$$\alpha =\frac{\lambda_{\text{PN}}-\lambda_{BN}}{\left(\lambda_{\text{PN}}-\lambda_{BN})+(\lambda_{BN}-\lambda_{PP}\right)}$$ (1)

$$\beta =\frac{\lambda_{\text{BN}}-\lambda_{NN}}{\left(\lambda_{\text{BN}}-\lambda_{NN})+(\lambda_{NP}-\lambda_{\text{PP}}\right)}$$ (2)

## 4 Experiment simulation

### 4.1 Experimental environment

The experimental environment uses a 64-bit Win 10 operating system, a quad-core, eight-thread Intel(R) Core (TM) i5-10210U CPU, and 8GB DDR4 RAM, and is programmed in PyCharm and Python 3.10.

### 4.2 Data set introduction

The data set used in this paper is CICIDS2017 [28] and DDoS SDN Data set [29]. The CICIDS2017 data set is from the cooperation project between the Communication Security Institution (CSE) and the Canadian Cyber Security Institute (CIC). The DDoS SDN Data set is from Bennett University and is a publicly available data set.

#### 4.2.1 CICIDS2017 data set.

The CICIDS2017 data set captures 5 days of data traffic and includes 8 CSV files, which are based on timestamp, source and destination ip, source and destination port, protocol, and attack. The data set includes both normal and attack traffic, with a total of 15 different traffic categories, including 1 normal traffic and 14 attack traffic, and contains 79 data characteristics. This paper mainly studies DDoS attacks, so we only select CSV files containing DDoS attacks on Friday afternoon for the experiment, and the data distribution is shown in Table 2.

**Table 2:** Data distribution of cicids2017 data set.

Label	Data	Total
***BENIGN***	97718	225745
***DDoS***	128027	

#### 4.2.2 DDoS SDN data set.

The DDoS SDN Data set is a collection of SDN-pecific data sets generated using the Mininet simulator, including benign TCP, UDP, and ICMP traffic as well as malicious traffic, TCP SYN attacks, UDP flood attacks, and ICMP attacks. There are 23 features in the data set, and the type distribution of the data set is shown in Table 3.

**Table 3:** Data distribution of DDoS SDN data set.

Label	Data	Total
***UDP***	16089	63561
***Attack data TCP***	15570	
***ICMP***	31902	
***UDP***	17499	40784
***Normal data TCP***	13866	
***ICMP***	9419	

### 4.3 Data set preparation

Data preprocessing is a series of processes on the original data set. Two data sets are used in this paper: the CICIDS2017 data set and the DDoS SDN Data set. The processing is roughly the same for both data sets. First, the original data set is read, the Numpy module is used to load the data, and then the attribute column of the data is de-duplicated. Then the whole data set is normalized, and the thermal coding operation is carried out. Then the high-dimensional data is reduced by PCA technology. Finally, the data dimensions are reorganized into two-dimensional matrix vectors, which are used as inputs to the ConvLSTM.

#### 4.3.1 CICIDS2017 data set preprocessing.

(1) Processing Duplicate Columns: When collecting statistics on the data set, two duplicate attribute columns are found. The attribute names are “Fwd Header Length”, and the sample attribute values are the same. Therefore, one of the attributes is deleted.
(2) Processing default values: There are default values in the columns “Flow Bytes/s” and “Flow Packets/s” in the data set. Its form is “infinity” and “NaN”, because the sample containing the default value is very small, so directly delete the sample containing the default value.
(3) Label normal data and attack data 0 and 1.
(4) Min-max normalization.

Min-max normalization is a common normalization method, which can normalize data into the interval [0, 1]. The normalization calculation method is shown in equation (3).


$$x^{\prime }=\frac{x-\min_{i}}{\max_{i}-\min_{i}}$$ (3)

Where: x is a value in the I-th attribute column; $min_{i}$ is the minimum value of the I-th attribute column; $max_{i}$ is the maximum value of the I-th attribute column.

(5) One-Hot Encoding.
(6) PCA technique reduces the dimension of the data set

PCA is one of the most widely used data dimensionality reduction algorithms. The main idea of PCA is to map N-dimensional features to K-dimensional features, which are new orthogonal features, also known as principal components, and reconstruct K-dimensional features on the basis of the original N-dimensional features.

Input: Data set $X=\left\{x_{1},x_{2},\ldots ,x_{n}\right\}$, down to the k dimension.

1) De-averaging (i.e., decentralization), in which each feature subtracts its average.
2) Calculate the covariance matrix $\frac{1}{n}XX^{T}$.
3) Eigenvalues and eigenvectors of covariance matrix $\frac{1}{n}XX^{T}$ are obtained by the eigenvalue decomposition method.
4) Sort the eigenvalues from largest to smallest, choosing the largest k  of them. Then the corresponding  k eigenvectors are used as row vectors to form the eigenvector matrix P.
5) Transform the data into a new space constructed by k feature vectors, I, e. *Y = PX*.
6) Data dimension reorganization.

The input data in the ConvLSTM network is a two-dimensional matrix, so the processed data needs to be reshaped into a two-dimensional matrix using the Reshape function. When reshaping the matrix, 0 is added at the end of the matrix to solve the conflict between dimension and matrix elements.

#### 4.3.2 DDoS SDN data set data preprocessing.

(1) Handling Default Values: The rx_kbps and tot_kbps columns in the data set contain default values due to the default sample size being less so just delete it. (2) Min-max normalization. (3) One-Hot Encoding. (4) PCA technique reduces the dimension of the data set. (5) Data dimension reorganization.

### 4.4 Experimental preparation

#### 4.4.1 Evaluation index.

The evaluation indicators in this experiment are evaluated based on accuracy(ACC), Precision(PR),false positive rate(FPR), recall rate(Re), and F1 score, and each evaluation indicator is calculated from the following equation. TP is true positive, FP is false positive, TN is true negative, and FN is false negative. The calculation method is shown in equations (4)-(8).


$$\text{A}ccuracy=\frac{TP+TN}{TP+FP+FN+TN}$$ (4)

$$\mathrm{Pr}\text{e}cision=\frac{TP}{TP+FP}$$ (5)

$$\text{FPR}=\frac{FP}{FP+NP}$$ (6)

Recall=TPTP+NP (7)

F1=2*Precision*RecallPrecision+Recall 8)

#### 4.4.2 Sample selection and parameter setting.

In the experiment, the pre-processed data set was randomly divided into 8:2, with 80% of the data set as the training set and 20% as the test set. The parameters of the three-way decisions have been given, and the remaining parameters of the ConvLSTM-TWD model are shown in Table 4.

**Table 4:** Parameter Settings for ConvLSTM.

Name	Parameter
***ConvLSTM***	Filters = 64，kernel_size=(1, 3)
***MultiHeadAttention***	num_heads = 4，key_dim = 64
***CICIDS2017learning_rate***	0.0001
***DDoS SDN Data set learning_rate***	0.001

### 4.5 Experimental results and analysis

#### 4.5.1 Hyperparameter experiment.

**Fig 4:**
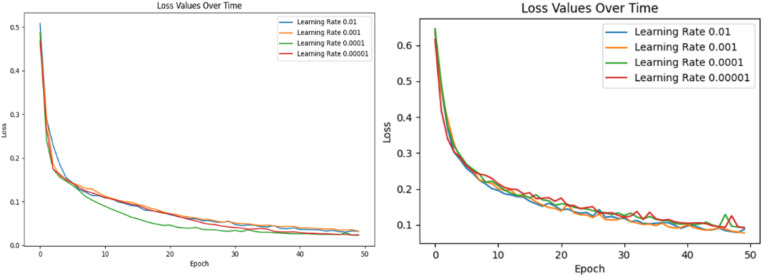
Relationship between loss value and learning rate on CICIDS2017 and DDoS SDN data sets.

This paper conducted experiments on the CICIDS2017 data set and the DDoS SDN Data set respectively for the changes in loss function values when different learning rates were used and obtained results as shown in Tables 5 and 6 and Fig 4. Table 5 shows that when the learning rate gradually decreases from 0.01, the evaluation indicator values gradually improve. When it decreases to 0.0001, they reach their best, and then the evaluation indicator values gradually deteriorate. Also, the left part of Fig 4 shows that when the learning rate is 0.0001, the smallest loss function value is obtained during training on CICIDS2017 data set. So the learning rate of the experiment is set to 0.0001. Similarly, When the learning rate was 0.001, the evaluation indicator values are best on SDN Data set, and the smallest loss function value during training, so the learning rate of the experiment was set to 0.001.

**Table 5:** Hyperparameter experiments of CICIDS2017 data set.

learning_rate	ACC	Re	FPR	PR	F1
***0.01***	0.980	0.971	0.012	0.983	0.977
***0.001***	0.988	0.984	0.007	0.989	0.987
***0.0001***	**0.994**	**0.990**	**0.003**	**0.995**	**0.993**
***0.00001***	0.988	0.978	**0.003**	**0.995**	0.986

**Table 6:** Hyperparameter experiment of DDoS SDN data set.

learning_rate	ACC	Re	FPR	PR	F1
***0.01***	0.952	**0.981**	0.066	0.905	0.942
***0.001***	**0.977**	0.965	**0.015**	**0.975**	**0.970**
***0.0001***	0.938	0.917	0.048	0.924	0.920
***0.00001***	0.954	0.941	0.037	0.942	0.941

**Fig 5:**
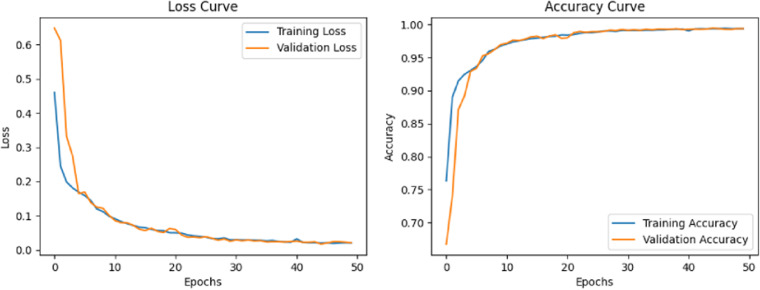
Loss and Accuracy rates of ConvLSTM-MHA-TWD on CICIDS2017 data set.

**Fig 6:**
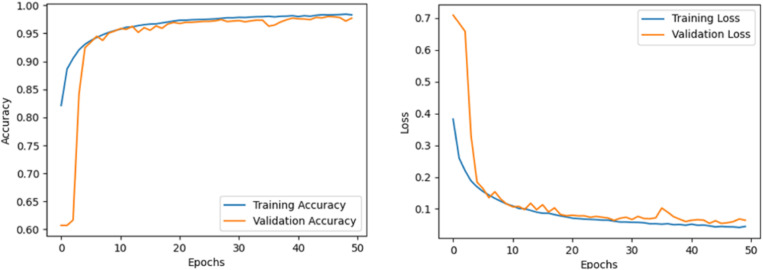
Accuracy and loss rate of ConvLSTM- MHA-TWD on DDoS SDN data set. (a) Loss Curve, (b) Accuracy Curve.

During the training of the model in this paper, we iterated 50 times on the CICIDS2017 data set, and the experimental results obtained are shown in Fig 5. According to the experimental data, the accuracy of the model is 0.994 and the loss rate is 0.02. For the DDoS SDN Data set, we also did 50 iterations of model training to get the best effect, in which the accuracy rate was 0.977 and the loss rate was 0.07, as shown in Fig 6.

#### 4.5.2 Ablation experiment.

Two types of comparison experiments were designed. The first type of ablation experiment evaluated the effectiveness of ConvLSTM-MHA-TWD’s ConvLSTM, multi-head attention mechanism and three-way decision-making. The ablation experiments included ConvLSTM, ConvLSTM-MHA, ConvLSTM-TWD and ConvLSTM-MHA-TWD, and the experimental results were shown in Tables 7 and 8. According to the analysis of the experimental results, ConvLSTM can achieve a good effect in the feature extraction of data and enhance the performance of the model after adding a multi-head attention mechanism. MHA can help the model effectively capture the patterns and relationships in the data. It can be seen that ConvLSTM has certain advantages in extracting spatiotemporal features, and TWD can effectively reduce data misclassification and improve the accuracy of intrusion detection.

**Table 7:** Ablation results of CICIDS2017 data set.

Model	ACC	Re	FPR	PR	F1
***ConvLSTM-MHA-TWD***	**0.994**	**0.990**	**0.003**	**0.995**	**0.993**
***ConvLSTM-TWD***	0.971	0.941	0.005	0.992	0.966
***ConvLSTM-MHA***	0.958	0.960	0.019	0.942	0.951
***ConvLSTM***	0.938	0.883	0.044	0.972	0.925

**Table 8:** Ablation results of DDoS SDN data set.

Model	ACC	Re	FPR	PR	F1
***ConvLSTM-MHA-TWD***	0.977	0.965	0.015	0.975	0.970
***ConvLSTM-TWD***	0.959	0.938	0.026	0.957	0.947
***ConvLSTM-MHA***	0.934	0.915	0.053	0.916	0.915
***ConvLSTM***	0.926	0.896	0.054	0.914	0.905

In the model we use, the batch_size value is set to 1000, the epochs value is 50, the optimizer is the Adam optimization algorithm, and the loss function is binary cross entropy [30].

**Fig 7:**
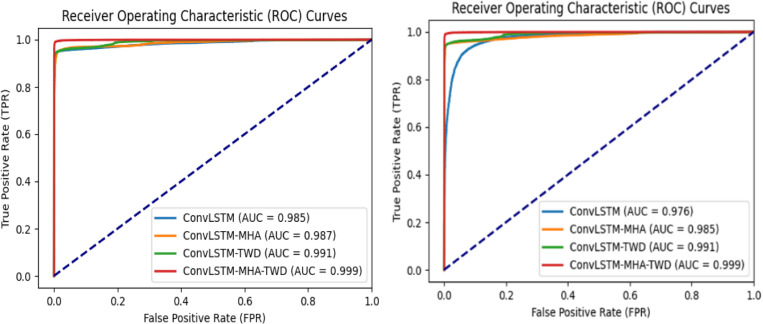
ROC curve comparison on CICIDS2017 and DDoS SDN data sets.

Fig 7 shows the comparison of ROC curves during ablation. The ROC curve is also known as the sensitivity curve. The more convex the ROC curve is to the upper left, the better the classification performance is. AUC is the area under the ROC curve used to quantify classifier performance. The value of AUC ranges from 0 to 1, with higher values indicating better classifier performance. As can be seen from the figure, the ROC curve area of the model proposed in this paper achieved the maximum value in the experiments on the two data sets, indicating that the model presented in this paper has the best performance.

#### 4.5.3 Contrast experiment.

The second type of comparison experiment uses the CICIDS2017 data set and the DDoS SDN Data set to get experimental results. In the case of the same data set, the model in the table is reproduced compared with the original text and the experiment is carried out, and the experimental results achieved are not as good as the experimental results of the model proposed in this paper. The model used in this paper has a higher accuracy, recall rate, accuracy rate, and F1 score than the comparison model. It can be concluded that the proposed model has high robustness in feature extraction and classification of intrusion detection data.

**In Experiment 1,** random forest (RF), k -nearest neighbor (KNN), support vector machine (SVM), and Bayesian model (BYS) were selected as the comparison models based on the three-way decision classification method, and the feature extraction method was based on ConvLSTM. Experiments were carried out on the CICIDS2017 data set and the DDoS SDN Data set respectively, and the experimental results are shown in Tables 9 and 10.

**Table 9:** Comparison of experimental results of different classification models in CICIDS2017 data set.

Model	ACC	Re	FPR	PR	F1
***ConvLSTM-MHA-TWD***	**0.994**	**0.990**	**0.003**	**0.995**	**0.993**
***ConvLSTM-RF***	0.913	0.898	0.074	0.900	0.899
***ConvLSTM-KNN***	0.939	0.920	0.045	0.938	0.929
***ConvLSTM-SVM***	0.947	0.929	0.039	0.947	0.938
***ConvLSTM-BYS***	0.923	0.909	0.065	0.912	0.911

**Table 10:** Comparison of experimental results of different classification models in the DDoS SDN data set.

Model	ACC	Re	FPR	PR	F1
***ConvLSTM- MHA-TWD***	**0.977**	**0.965**	**0.015**	**0.975**	**0.970**
***ConvLSTM-RF***	0.762	0.708	0.203	0.692	0.700
***ConvLSTM-KNN***	0.764	0.684	0.183	0.707	0.695
***ConvLSTM-SVM***	0.822	0.760	0.137	0.782	0.771
***ConvLSTM-BYS***	0.823	0.740	0.122	0.795	0.767

As can be seen from the experimental results in Tables 10 and 11, the TWD-based classification model is superior to the results obtained by other classification models inevaluation indicators. In particular, the false positive rate is significantly lower than other models, which indicates that the three-way decision-based classification model proposed in this paper is superior to other classification algorithms in comprehensive performance. The introduction of a boundary domain to traditional two-way decisions can avoid the risk of misclassification of uncertain data and greatly improve the accuracy of intrusion detection. The application of three-way decision theory has a positive impact in the field of intrusion detection.

**Table 11:** Experimental comparison results of different feature extraction models on CICIDS2017 data set.

Model	ACC	Re	FPR	PR	F1
***ConvLSTM- MHA-TWD***	**0.994**	**0.990**	**0.003**	**0.995**	**0.993**
***PCA-TWD***	0.926	0.934	0.079	0.898	0.916
***DNN-TWD***	0.945	0.886	0.010	0.984	0.932
***FA-TWD***	0.913	0.857	0.043	0.937	0.895
***SVD-TWD***	0.902	0.842	0.051	0.924	0.881

**Fig 8:**
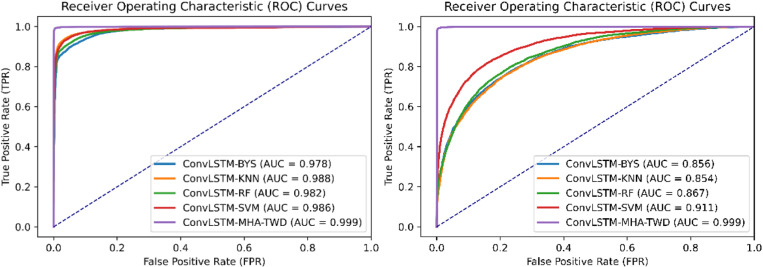
ROC curves for different classifiers on CICIDS2017 and DDoS SDN data sets.

Fig 8 shows ROC curves of different methods on the data set CICIDS2017 and DDoS SDN Data set respectively. According to the figure, the AUC area of the ConvLSTM-MHA-TWD model is the largest, which proves that the ConvLSTM-MHA-TWD model has better comprehensive performance.

**In experiment 2,** Principal component analysis (PCA), deep neural network (DNN), factor analysis (PA), singular value decomposition (SVD), and ConvLSTM were selected for comparison to verify the effect of ConvLSTM feature extraction based on the three-way decision classification algorithm. Different results obtained by experiments on the CICIDS2017 data set and the DDoS SDN Data set are shown in Tables 11 and 12.

**Table 12:** Experimental comparison results of different feature extraction models in the DDoS SDN data set.

Model	ACC	Re	FPR	PR	F1
***ConvLSTM- MHA-TWD***	**0.977**	**0.965**	**0.015**	**0.975**	**0.970**
***PCA-TWD***	0.889	0.845	0.081	0.870	0.857
***DNN-TWD***	0.925	0.905	0.060	0.905	0.905
***FA-TWD***	0.941	0.927	0.049	0.923	0.925
***SVD-TWD***	0.861	0.787	0.090	0.849	0.817

As can be seen from the experimental results in Tables 11 and 12, ConvLSTM-MHA-TWD, the algorithm proposed in this paper, has obtained the best results among the five evaluation indexes, and its comprehensive performance is significantly better than other algorithms. The main reason is that our model can obtain more comprehensive features by combing ConvLSTM and MHA to form a residual connection. It can be concluded that the data features obtained through ConvLSTM map better to the original data.

**Fig 9:**
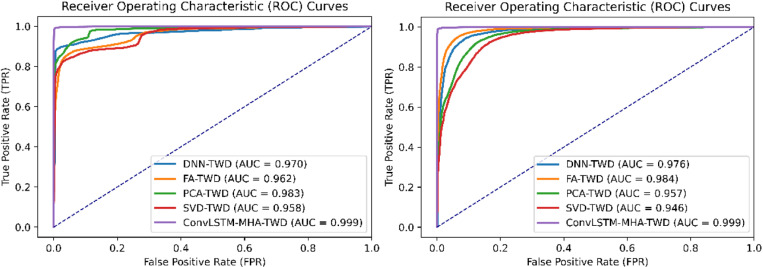
ROC curves of different feature extraction models on CICIDS2017 and DDoS SDN data sets.

Fig 9 are ROC curve comparison graphs of different methods on the CICIDS2017 data set and the DDoS SDN Data set respectively. The ROC curve graph shows that the ConvLSTM-MHA-TWD model has the largest AUC area. It is proved that the ConvLSTM-MHA-TWD model has better overall performance.

**Experiment 3,** This experiment is to explore the performance comparison between the ConvLSTM-TWD model proposed in this paper and other models. The comparison models selected for the experiment in this paper include: DDoS attack detection scheme based on Bi-LSTM (BiLSTM) [23], intrusion detection method based on a heuristic optimization algorithm and swarm intelligence algorithm combined with short-short-memory network (HHO-PSO-LSTM) [31], intrusion detection algorithm based on convolutional neural network and bidirectional short-short-memory network(CNN-BiLSTM) [32], intrusion deection method based on gated cyclic unit and support vector machine (GRU-SVM) [33], intrusion detection algorithm based on convolutional neural network and three-way decision making (CNN-TWD) [34], Intrusion detection algorithm based on convolutional neural network and bidirectional short-short-memory network and three-way decision making (CNN-BiLSTM-TWD) [35]. Experiments were conducted on the CICIDS2017 data set and the DDoS SDN Data set respectively. As shown in Tables 13 and 14, the detection results of the proposed algorithm were compared with those of other algorithms under the same experimental environment. As can be seen from Tables 13 and 14, the ConvLSTM-MHA-TWD DDoS intrusion detection model proposed in this paper outperforms the comparison algorithm in four indexes: accuracy (ACC), detection rate (DR), false positive rate (FPR), F1 score (F1). From the comprehensive analysis, the comprehensive performance of the ConvLSTM-MHA-TWD method proposed in this paper is better than other comparative intrusion algorithms. Because ConvLSTM-MHA-TWD processing features by convolution and long short-term feature processing, costs more time than other method.

**Table 13:** Comparison of experimental results of different algorithms in CICIDS2017 data set.

Model	ACC	Re	FPR	PR	F1	Time/s
***ConvLSTM- MHA-TWD***	**0.994**	**0.990**	**0.003**	**0.995**	**0.993**	736.43
***CNN-TWD*** [34]	0.957	0.956	0.042	0.944	0.950	561.20
***CNN-BiLSTM-TWD*** [35]	0.986	0.978	0.007	0.989	0.984	752.82
***GRU-SVM*** [33]	0.910	0.821	0.022	0.964	0.887	635.90
***HHO-PSO-LSTM*** [31]	0.915	0.851	0.035	0.947	0.896	869.66
***BiLSTM*** [23]	0.903	0.824	0.036	0.944	0.880	**521.60**
***CNN-BiLSTM*** [32]	0.959	0.921	0.011	0.984	0.951	687.30

**Table 14:** Comparison of experimental results of different algorithms in the DDoS SDN data set.

Model	ACC	Re	FPR	PR	F1	Time/s
***ConvLSTM- MHA-TWD***	**0.977**	**0.965**	**0.015**	**0.975**	**0.970**	435.69
***CNN-TWD*** [34]	0.953	0.938	0.037	0.942	0.940	**316.54**
***CNN-BiLSTM-TWD*** [35]	0.957	0.926	0.022	0.963	0.944	501.28
***GRU-SVM*** [33]	0.847	0.896	0.184	0.758	0.821	407.18
***HH0-PSO-LSTM*** [31]	0.848	0.924	0.200	0.749	0.827	583.75
***BILSTM*** [23]	0.857	0.893	0.165	0.776	0.831	358.73
***CNN-BiLSTM*** [32]	0.947	0.934	0. 040	0.937	0.935	420.60

**Fig 10:**
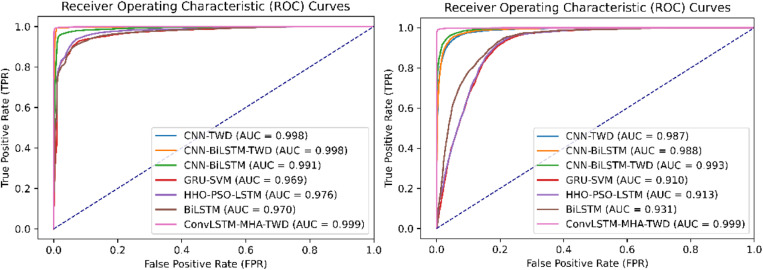
ROC curves of different comparison experiments on CICIDS2017 and DDoS SDN data sets.

Fig 10 shows the ROC curve comparison diagram of different intrusion detection algorithms in the CICIDS2017 data set. It can be seen from the graph that the ConvLSTM-MHA-TWD model proposed in this paper has the largest AUC area value, which proves that the algorithm is more comprehensive. Fig 10 shows the ROC curve comparison of different intrusion detection algorithms in the DDoS SDN Data set. Although the area of the ROC curve of the CNN-BiLSTM-TWD model is slightly larger than the model proposed in this paper, the model proposed in this paper is higher than the comparison model in other performance evaluation indicators. Therefore, the intrusion detection performance of the proposed model is good.

## 5 Conclusions

In view of the complexity of network security problems in today’s big data environment, the feature extraction effect of intrusion detection data in the network is not good, and the gradient disappearance problem is prone to occur when using multi-layer neural networks, and the misclassification of data occurs. In this paper, an intrusion detection algorithm, ConvLSTM-MHA-TWD, which is based on an improved convolutional long- and short-time memory fully connected neural network and three-way decision-making, plays an active role in solving this problem. ConvLSTM’s convolution and short-and long-time features are used to extract the features of input data in parallel, and the multi-head attention mechanism is used to further focus on the dependency between different features to improve the feature extraction efficiency. The residual connection is used to prevent the gradient disappearance caused by too many layers of the model, ensure the stability and detection effect of the model training process, and improve the feature extraction ability of the network. The three-way decision theory can effectively reduce the misclassification of data and improve the accuracy of intrusion detection.

This method is implemented based on the data sets CICIDS2017 and DDoS SDN, which are suitable for training large amounts of data, but it takes a long time to train model. How to solve the problem and apply it in an SDN environment is our future work.

## Supporting information

S1 File Data. (ZIP)
